# Planning and implementing practice changes in Ontario maternal-newborn hospital units: a secondary qualitative analysis

**DOI:** 10.1186/s12884-023-06042-1

**Published:** 2023-10-17

**Authors:** Jessica Reszel, Olivia Daub, Sandra I. Dunn, Christine E. Cassidy, Kaamel Hafizi, Marnie Lightfoot, Dahlia Pervez, Ashley Quosdorf, Allison Wood, Ian D. Graham

**Affiliations:** 1 School of Nursing, University of Ottawa, 200 Lees Avenue, Ottawa, ON K1N 6N5 Canada; 2 Clinical Epidemiology Program, Ottawa Hospital Research Institute, 501 Smyth Road, Ottawa, ON K1H 8L6 Canada; 3 Better Outcomes Registry & Network (BORN) Ontario, 401 Smyth Road, Ottawa, ON K1H 8L1 Canada; 4 School of Communication Sciences and Disorders, Western University, 1201 Western Road, London, ON N6G 1H1 Canada; 5 School of Nursing, Dalhousie University, 5869 University Avenue, Halifax, NS B3H 4R2 Canada; 6 IWK Health Centre, 5980 University Avenue, Halifax, NS B3K 6R8 Canada; 7 Women and Children’s Health Network, Orillia Soldiers’ Memorial Hospital, 170 Colborne St W, Orillia, ON L3V 2Z3 Canada; 8 Parent Research Advisor, Ottawa, ON Canada; 9 Neonatal Intensive Care Unit, The Ottawa Hospital, 501 Smyth Road, Ottawa, ON K1H 8L6 Canada; 10 School of Epidemiology and Public Health, University of Ottawa, 600 Peter Morand Crescent, Ottawa, ON K1G 5Z3 Canada

**Keywords:** Maternal-newborn care, Practice changes, Implementation science, Implementation practice, Qualitative secondary analysis

## Abstract

**Background:**

Moving evidence into practice is complex, and pregnant and birthing people and their infants do not always receive care that aligns with the best available evidence. Implementation science can inform how to effectively move evidence into practice. While there are a growing number of examples of implementation science being studied in maternal-newborn care settings, it remains unknown how real-world teams of healthcare providers and leaders approach the overall implementation process when making practice changes. The purpose of this study was to describe maternal-newborn hospital teams’ approaches to implementing practice changes. We aimed to identify what implementation steps teams take (or not) and identify strengths and potential areas for improvement based on best practices in implementation science.

**Methods:**

We conducted a supplementary qualitative secondary analysis of 22 interviews completed in 2014–2015 with maternal-newborn nursing leaders in Ontario, Canada. We used directed content analysis to code the data to seven steps in an implementation framework (Implementation Roadmap): identify the problem and potential best practice; assemble local evidence; select and customize best practice; discover barriers and drivers; tailor implementation strategies; field-test, plan evaluation, prepare to launch; launch, evaluate, and sustain. Frequency counts are presented for each step.

**Results:**

Participants reported completing a median of 4.5 of 7 Implementation Roadmap steps (range = 3–7), with the most common being identifying a practice problem. Other steps were described less frequently (e.g., selecting and adapting evidence, field-testing, outcome evaluation) or discussed frequently but not optimally (e.g., barriers assessment). Participants provided examples of how they engaged point-of-care staff throughout the implementation process, but provided fewer examples of engaging pregnant and birthing people and their families. Some participants stated they used a formal framework or process to guide their implementation process, with the most common being quality improvement approaches and tools.

**Conclusions:**

We identified variability across the 22 hospitals in the implementation steps taken. While we observed many strengths, we also identified areas where further support may be needed. Future work is needed to create opportunities and resources to support maternal-newborn healthcare providers and leaders to apply principles and tools from implementation science to their practice change initiatives.

**Supplementary Information:**

The online version contains supplementary material available at 10.1186/s12884-023-06042-1.

## Background

Improving the quality of care in maternal-newborn settings is an international, national, and local priority [1–3]. Despite ongoing improvement efforts, pregnant and birthing people and their infants continue to receive care that is not always aligned with the best available evidence [4, 5]. Moving evidence into practice is complex and remains an ongoing challenge in healthcare. Studies have revealed that individuals and organizations face many barriers to implementing evidence into practice, including lack of skills with few opportunities for training, unsupportive organizational cultures and the undervaluing of evidence, as well as limited time and physical and human resources [6]. In maternal-newborn care specifically, clinical practice changes can be particularly complex due to the involvement of different healthcare providers (e.g., nurses, physicians, midwives), care that focuses on two different patient populations (e.g., the pregnant or birthing person and infant), and the fact that some practices are affected by separate hospital units (e.g., birthing unit, mother-baby unit, neonatal intensive care unit).

Implementation science is defined as “the scientific study of methods to promote the systematic uptake of research findings and other evidence-based practices into routine practice” [7]. Although the field of implementation science has developed rapidly over the past two decades, its full potential has not yet been realized [8]. There is emerging discussion about how the knowledge gained through implementation science has largely remained in the scientific domain, and has not been well translated into practice-based settings such as healthcare [9, 10]. It is important to assess if and how implementation evidence, principles, and tools are being applied to identify opportunities to optimize evidence-informed implementation.

Within maternal-newborn care, there are increasing examples of implementation science applications, focusing on topics such as prioritizing content areas for implementation research [11] and practice [12], identifying and examining the effectiveness of different implementation strategies [13–15], and exploring barriers and facilitators to implementation [15–17]. While this literature makes an essential contribution to advancing our understanding of evidence-informed strategies to implement evidence and the potential challenges, it typically has not focused on the entire implementation process needed to bring about change (i.e., taking a planned action [18] approach to changes). Recently, there have been more examples of how teams work through the overall implementation process being published as best practice implementation reports [19–21]. However, these reports are typically focused on a practice change in a single setting, and by virtue of publishing their work, likely over-represent teams that are more familiar (and potentially more successful) with implementation processes. To complement this existing literature, there is a need to shift from learning about single implementation strategies or single projects to also looking more holistically at how maternal-newborn teams implement practice changes in their day-to-day work.

The province of Ontario, Canada provides a unique opportunity to learn about the process of moving evidence into practice. Every birthing hospital in the province has access to a perinatal data registry called the Better Outcomes Registry & Network (BORN) Ontario, which captures data on nearly every birth in the province [22]. Contributing hospitals can use their own BORN data to facilitate practice improvements [22], for example, to identify evidence-practice gaps, understand current practice, and monitor and evaluate implementation projects. Although hospitals have access to this large and robust data system, it remains largely unknown what processes teams are using to implement practice changes and how well their processes align with current best practices in implementation science.

In 2012, BORN launched the Maternal Newborn Dashboard (“the dashboard”), which is an online audit and feedback system that maternal-newborn hospital teams can use to facilitate practice change improvements. The dashboard includes six key performance indicators related to practices such as newborn screening, episiotomies, breastfeeding, repeat elective cesarean sections, Group B streptococcus screening, and inductions [23]. In 2014, an evaluation of the dashboard commenced, providing an opportunity to learn how Ontario maternal-newborn hospitals approach practice changes and how they use the dashboard to support their work. One part of the evaluation involved interviews with nursing leaders in Ontario maternal-newborn hospitals about how they implement practice changes.

Using these data, we aimed to understand maternal-newborn leaders’ usual approaches to implementing practice changes in their hospital units, including what steps they take or not, and identify potential areas where the implementation process could be improved.

## Methods

While the focus of this current paper is to present the results of a qualitative secondary analysis, as per the reporting guidance of Beck [24], we report the methods of the primary study for context, as well as the methods for this current secondary analysis. We used the Consolidated Criteria for Reporting Qualitative Research checklist (Additional file 1) to guide our reporting [25].

### Methods for previously conducted primary study

The methods for the primary study, which was one part of a larger mixed-methods evaluation, were detailed in a published protocol [23] and are summarized below.

#### Objectives

The objective of the primary study was to qualitatively explore potential factors that may explain the differences among maternal-newborn hospitals in their use of the dashboard. Because the purpose of the interviews was to inform the development of a questionnaire for all Ontario maternal-newborn hospitals to measure the identified factors, the interview data were never prepared for publication.

#### Design

The primary study used a qualitative descriptive design [26].

#### Sample

A criterion-based approach [27] was used to identify a purposeful sample of obstetrical managers and directors at maternal-newborn hospitals in Ontario, Canada, reflecting different birth volumes, acuity levels, geographic locations, and engagement with the dashboard. Obstetrical managers and directors were targeted due to their knowledge of clinical practice, quality improvement processes, and dashboard use at their organization. A total of 34 individuals were invited by email: three declined participation, nine did not respond, and 22 consented. The researchers assessed for data saturation throughout recruitment, data collection, and analysis. Recruitment stopped when interviews did not lead to new information [28].

#### Data collection

The original research team developed a semi-structured interview guide (Additional file 2) informed by the Promoting Action on Research Implementation in Health Services (PARIHS) framework [29], the Organizational Readiness for Knowledge Translation (OR4KT) tool [30, 31], and the Knowledge-to-Action framework [32]. The interviews were conducted between November 2014 and March 2015 by one of two female research staff (master’s-prepared research coordinator with expertise in quality improvement; research assistant with maternal-newborn nursing experience). Both interviewers had qualitative research experience and were trained by the study investigators. The interviewers did not have a prior relationship with study participants. The interviews, which lasted an average of 34 min (range of 17 min to 49 min), were completed by telephone and audio-recorded. Interviews were transcribed verbatim by a transcriptionist and verified by the research team.

### Methods for current secondary analysis of primary study

#### Objectives

The objectives of this current secondary analysis were to: (1) describe maternal-newborn teams’ approaches to implementing practice changes; (2) identify the implementation steps and activities that teams do and do not take; and (3) identify any strengths and potential areas for improvement based on best practices in implementation science.

#### Design

The study we report here is a supplementary analysis [33]. Specifically, we conducted a more detailed analysis of one component of the dataset (i.e., approaches to implementing practice changes) that was not the focus of the primary study [33].

#### Researchers’ relationship to the primary dataset

The lead researcher for this secondary analysis (Reszel) was a research coordinator for the overall evaluation study [23], but was not directly involved in the collection or analysis of the primary dataset. The co-principal investigator (Dunn) and a co-investigator (Graham) from the primary study were involved in this secondary analysis and provided contextual and methodological details as needed.

#### Data used

We obtained permission from the co-principal investigator of the primary study (Dunn) and research ethics board approval to access the de-identified transcripts. Aggregate demographic information was provided for contextual information. We did not collect any new supplementary data.

#### Assessment of quality and fit of dataset for secondary analysis

Of the 22 interviews, 21 transcripts were available. There was no transcript for one interview due to the audio-recorder malfunctioning; however, detailed notes from this interview were available. There were specific questions in the semi-structured interview guide focused on participants’ usual practice change processes (questions 6–11 in Additional file 2), providing a rich dataset to assess if and how these practice change processes aligned with best practices in implementation science.

#### Data analysis

**Fig. 1:**
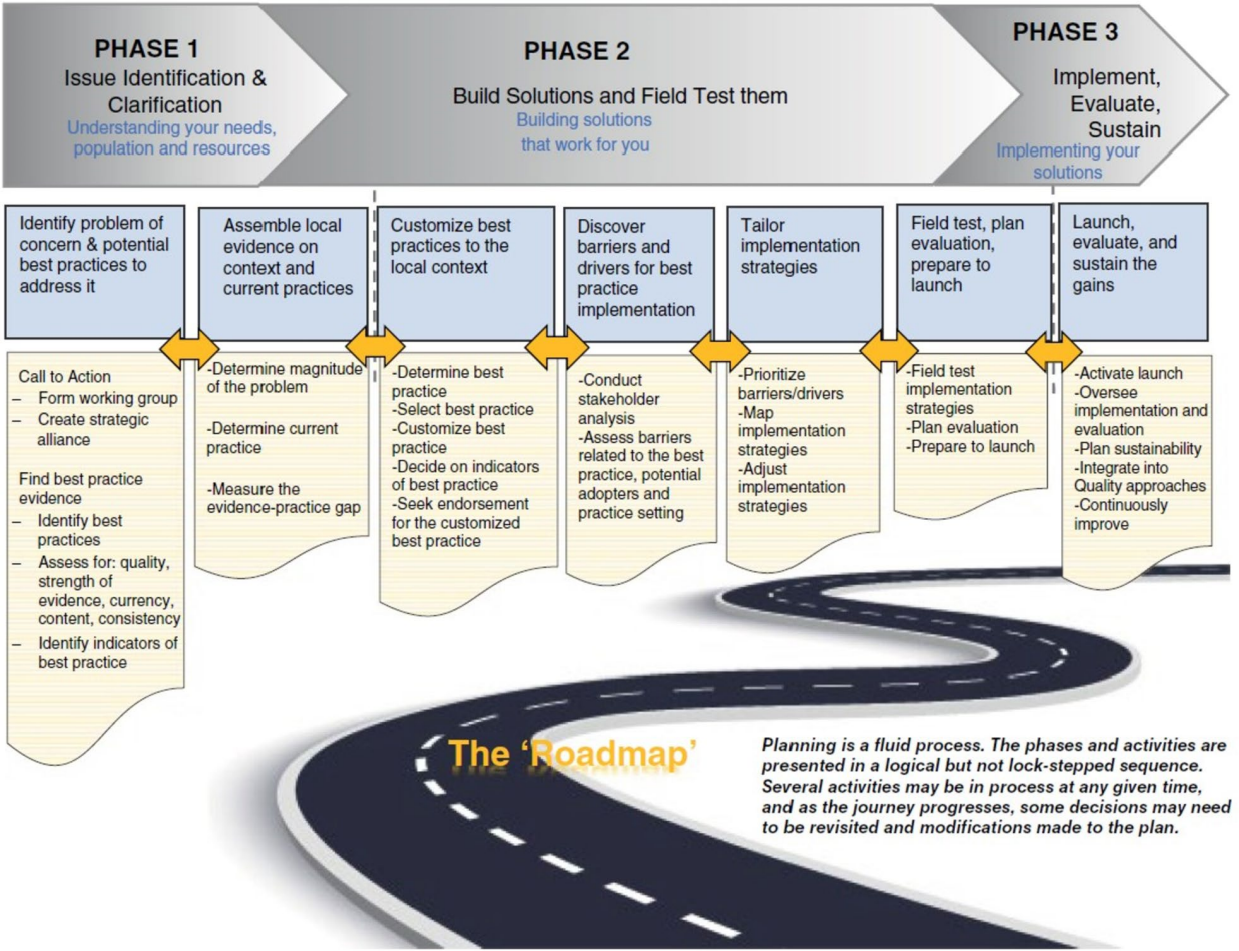
The Implementation Roadmap Copyright 2021 Wiley. Used with permission from Harrison MB, Graham ID, Knowledge Translation in Nursing and Healthcare: A Roadmap to Evidence-Informed Practice, John Wiley & Sons Inc [36]

We conducted a directed content analysis [34], using NVivo12 Pro for data management [35]. We used an implementation framework, the Implementation Roadmap [36], as the initial coding scheme. As a “planned action” framework [18], the Implementation Roadmap provides comprehensive guidance to facilitate implementation, presenting the necessary steps and activities to effectively plan and execute implementation projects [36]. It was therefore anticipated that coding according to the Implementation Roadmap would provide insight into the comprehensiveness of the teams’ approaches and highlight which steps were being taken or not. The initial coding scheme included the seven steps in the Implementation Roadmap, namely: (1) identify the problem of concern and potential best practice; (2) assemble local evidence on context and current practices; (3) select and customize best practice to local context; (4) discover barriers and drivers for best practice implementation; (5) tailor implementation strategies; (6) field-test, plan evaluation, prepare to launch; and (7) launch, evaluate, and sustain the gains (Fig. 1).

Each transcript was read in its entirety and coded by one team member (Reszel). Text was coded to the Implementation Roadmap steps where possible and frequency counts presented. Any content that could not be coded to the Implementation Roadmap was assigned a new code. For instance, we coded examples of engagement using the applicable levels described by Manafò et al. (e.g., inform, consult, involve, collaborate) [37]. The codes were grouped into broader categories. The coder (Reszel) met weekly with one other team member (Graham), who was the developer of the Implementation Roadmap, to discuss the coding and emerging trends. Once coding was completed, another team member (Daub), who has expertise in knowledge translation and implementation, reviewed all coded transcripts for accuracy and comprehensiveness. Throughout this review, the coders (Reszel, Daub) met to discuss the coding and categories and to reach consensus. The coding of the transcripts was subsequently updated. The coding review did not result in any changes to the coding scheme.

Finally, a summary of the analysis was presented to and discussed by the broader research team, which included a parent representative (Pervez), clinicians (Cassidy, Dunn, Lightfoot, Quosdorf), hospital intermediaries (Hafizi, Wood), and implementation science experts (Cassidy, Dunn). This discussion served to confirm and challenge the analysts’ interpretation and informed the final presentation of results.

#### Author positionality

Before presenting the results, we briefly describe our positionality. Our team includes healthcare providers and leaders, pregnant people and parents, intermediaries, and researchers of maternal-newborn care, with many of us having experience in multiple domains. We believe that the care provided to pregnant and birthing people should be informed by the best available evidence. We recognize it can be challenging to implement evidence-informed practices, but we believe that these challenges can be overcome and that maternal-newborn teams should be supported to develop the knowledge and skills to apply evidence in practice. This position influenced the question we chose to investigate and our professional and lived experiences informed the interpretation of our findings.

## Results

Participants came from diverse hospital settings in Ontario, representing different professional roles, levels of care, and birth volumes (Table 1).

**Table 1:** Demographic information of interview participants (*N* = 22)

Characteristic	n (%)
**Participant role**	
Clinical Manager	10 (45)
Director or Senior Leader	8 (36)
Quality Improvement Lead (RN)	3 (14)
Clinical Nurse Specialist	1 (5)
**Hospital level of maternal-newborn care**^a^	
Low risk (level 1)	6 (27)
Moderate risk (level 2)	13 (59)
High risk (level 3)	3 (14)
**Number of births per year**	
≤ 500	5 (23)
501–2499	7 (32)
≥ 2500	10 (45)

^a^As per the Provincial Council for Maternal and Child Health (PCMCH) Perinatal, Birthing and Newborn Levels of Care Guidance Document [38]

**Fig. 2:**
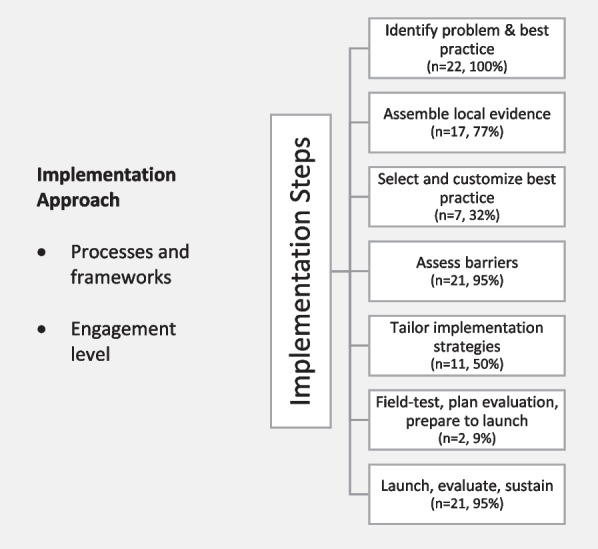
Organization of study findings

We present our results under two main categories: (1) implementation steps, including the seven steps in Implementation Roadmap [36], and (2) implementation approach, including processes/frameworks and engagement level (Fig. 2).

### Implementation steps

**Fig. 3:**
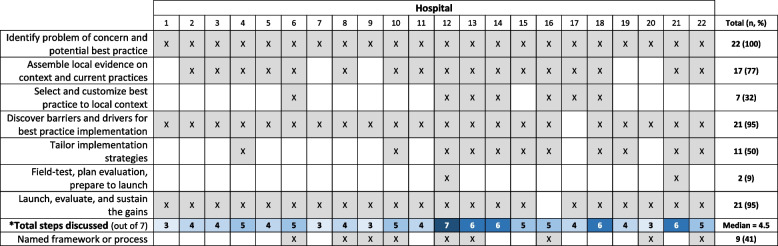
Implementation steps by hospital site *Discussed does not mean it was done optimally; but there was at least a general mention of the step

Across the sites, there were examples of each Implementation Roadmap step (Fig. 3). The most frequently described steps were identifying the problem and potential best practice (*n* = 22, 100%); assembling local evidence (*n* = 17, 77%); and identifying barriers and drivers (*n* = 21, 95%). The least frequently described steps were selecting and customizing the best practice (*n* = 7, 32%) and field-testing, planning evaluations, and preparing to launch (*n* = 2, 9%). Participants described using a median of 4.5 out of the 7 implementation steps (range of 3 to 7). One participant mentioned all seven steps in their interview.

#### Identify problem of concern and potential best practice to address it

All participants (*n* = 22) described how their teams came to identify a potential practice problem that needed to be addressed. The problem was identified through provincial, regional, or organizational priorities or mandates; emerging evidence learned through conferences, publications, and guidelines; seeing a red signal on their dashboard; and through seeing how their practice rates compared to other hospitals:

“The thing that actually drove the change wasn’t the evidence, it wasn’t the talking or creating a need, it was ‘okay, here we are, we’re red [on the dashboard] and this is embarrassing’…now we’re being compared to other people and how we fit in and it wasn’t pretty.” (Participant 5)

About one-third of participants (*n* = 7, 32%) discussed how they identify potential best practices that could address the problem. Sources included research literature, clinical practice guidelines (e.g., Registered Nurses’ Association of Ontario [RNAO], Society of Obstetricians and Gynecologists of Canada [SOGC]), and evidence from obstetrical safety training programs (e.g., MORE^OB^).

Only one participant mentioned appraising the evidence underlying the potential best practice.

#### Assemble local evidence on context and current practices

Most participants (*n* = 17/22, 77%) gave at least one example of how they learn about their current practice and the context in which the practice is occurring. This assessment occurred by collecting data and team experiences and impressions. Participants mentioned local data sources such as the BORN data registry and the dashboard, abstracted data from decision support, chart audits, and informal discussions with staff.

“You have subjective impressions of ‘we have a problem here; we know we do too many social inductions.’ You get that subjective perspective from staff and physicians but what the data does is make it clear. There’s no arguing with: here’s how many you did, right?” (Participant 10)

Participants described how they used different data sources to “drill down” to specific cases and explore precipitating factors, leading to a more fulsome understanding of what may be driving current practice (e.g., patient demographics, specific healthcare providers, time of day, data entry).

Finally, two participants (9%) described conducting what would be considered a formal “gap-analysis” to measure the difference between current practice and the best practice they are targeting (i.e., the evidence-practice gap).

#### Select and customize best practices to local context

One-third of participants (*n* = 7/22, 32%) discussed how their teams select and customize the best practice for their setting. While no participants described a structured process for selecting the specific best practice to be implemented, five participants (23%) explained the importance of securing the support or endorsement of others. This support was achieved through sharing the evidence for the best practice in an understandable way, showing how the best practice aligns with provincial and regional priorities, and ongoing discussions to share and resolve concerns, as described by a participant:

“People don’t stay up at night trying to do things wrong, so helping them understand the rationale for *why*—taking that extra time to appraise the research and look at translating that so it’s in simple terms that they would be able to understand why. Sometimes telling them who else has already done it this way helps with the buy-in and engagement as well.” (Participant 6)

One participant described the challenge of working as an interprofessional team where different professions rely on and value different sources of evidence, highlighting the need for a tailored approach to build support for the selected best practice. Finally, only one participant described how they customized the best practice, providing the example of modifying the recommendation to make it more achievable in their setting. There were no examples of teams customizing the best practice to align with their context by explicitly considering the who, what, how, and when.

#### Discover barriers for best practice implementation

Nearly all participants (*n* = 21/22, 95%) stated they consider potential barriers to implementing the selected best practice. Participants generally described the barriers assessment process as informal, involving brainstorming among the working group and general discussions with the broader clinical team. Several participants stated they were just familiar with the “usual” barriers based on their previous experience, resulting in a barriers assessment not being repeated for the current practice change initiative.

Three participants (14%) described a more systematic approach to assessing barriers, detailing specific steps they take to identify barriers and the application of a framework:

“When you do your root cause analysis you always look at opportunities and barriers. So it’s a SWOT [Strengths, Weaknesses, Opportunities, Threats] analysis, right? What are we doing well? What are we not doing well? What can we improve on? And then what are we anticipating from a change perspective is going to be a barrier? Who do we have to engage to eliminate that?” (Participant 10)

No participants described assessing which barriers are feasible to address or prioritizing which barriers to target for the most impact.

#### Tailor implementation strategies

All participants (*n* = 22) provided examples of strategies they use to implement practice changes in their settings (i.e., implementation strategies). In total, participants identified 14 different implementation strategies, with the most frequently mentioned being meetings and discussion, interprofessional teamwork, and staff education, training, or coaching (Table 2).

**Table 2:** Implementation strategies used by study participants to implement practice changes

Implementation strategies	n (%)
Meetings and discussion	20 (91)
Interprofessional teamwork	18 (82)
Staff education, training, coaching	16 (73)
General communication and information sharing	15 (68)
Learning from other sites	15 (68)
Champions	14 (64)
Audit and feedback	11 (50)
Engaging quality improvement (QI) or implementation expert	9 (41)
Developing policies and guidelines	9 (41)
Service re-organization	9 (41)
Patient education and engagement	6 (27)
Relationship building	6 (27)
Reminders	6 (27)
Equipment	1 (5)

Half the participants (*n* = 11, 50%) indicated they take some steps to tailor the implementation strategies, either by tailoring them to identified barriers or by tailoring them to the local context. Five participants (23%) gave examples of how they consider the identified barriers when choosing which implementation strategies to use. For example, one participant described how they changed the days they were offering a specific clinical service to address an identified barrier to meeting best practice guidelines:

“A perceived barrier was that physicians couldn’t get a scheduled time for their cesarean section after 39 weeks so they were doing them earlier. We thought okay, that’s a barrier that we were really only doing elective sections from Monday to Friday. So we’re working at removing that barrier by doing a bit more planning with anesthesia to plan the cesarean section on a weekend.” (Participant 21)

Seven participants (32%) indicated that they take some steps to tailor the implementation strategies to the local context. For instance, some participants described how instead of “recreating the wheel” they looked to strategies used by other sites and adapted them to fit with their local culture and ways of doing things.

#### Field-test, plan evaluation, prepare to launch

Two participants (9%) described piloting or trialing their change initiatives prior to full-scale implementation. These “tests of change” were described as an important way to engage a core group of key supports and gather their feedback on what works and what does not. This information was then used to adjust the selected strategies prior to broader implementation:

“And then being willing to trial something rather than implement something and just say ‘this is how it is.’ So we’ve adapted to try an idea and then if it doesn’t work, adjust it to what we’ve learned from that experience.” (Participant 12)

No participants described developing an evaluation plan prior to implementation or completing a pre-launch assessment.

#### Launch, evaluate, and sustain the gains

Nearly all participants (*n* = 21, 95%) stated they engaged in some form of evaluation and/or sustainability activities. The most frequently described activity was monitoring adherence to the best practice. Eighteen participants (82%) described how they monitored their practice change initiative over time by using tools such as the BORN data registry and the dashboard to track changes in rates and colored signals. This monitoring data could be used to assess whether the implementation strategies were effectively bringing about the desired change, or if the strategies needed to be adjusted or boosted:

“The BORN data lets us know exactly where we’re sitting, how big the problem is, and when you can pull it month-to-month you can kind of get a glimpse as to: are we making any improvements in the interventions [best practices]? We rolled out a signed consent for formula supplementation—did that make any difference in our rates? The lunch and learns that we do around supplementation and around breastfeeding issues and techniques—are they making any difference?” (Participant 13)

Participants described how the monitoring results were shared with staff via meetings and posting on unit boards and communicated to leadership (e.g., division chiefs) and committees (e.g., quality and safety committees). However, some participants described the challenge of monitoring changes without access to timely data. Fewer participants described undertaking process evaluations or impact evaluations, with only one participant stating they also evaluate process indicators and patient outcomes.

Many participants (*n* = 16, 73%) stated that they were taking steps to ensure the sustainability of their practice changes. Examples of sustainability strategies included partnering with healthcare providers from the onset to secure their buy-in; making organizational changes that entrench the change in day-to-day work; ongoing monitoring for non-adherence; and maintaining ongoing communication with the team about the practice change. As described by one participant:

“We’re measuring it consistently and communicating that back to the clinicians: the physicians, the nurses, the midwives. What works well is if you measure it, people pay more attention to it. So that will definitely be one of our initiatives to make sure that it is sustained.” (Participant 15)

### Implementation approach

#### Processes and frameworks

Nine participants (41%) named at least one formal process or framework they used to guide their change process. These processes and frameworks included Lean (*n* = 5), Plan-Do-Study-Act (PDSA) or Plan-Do-Check-Act (PDCA) (*n* = 5), Baby Friendly Initiative (BFI) steps (*n* = 3), project management (*n* = 1), as well as theories such as adult learning theory (*n* = 1) and change management theory (*n* = 1). One participant described the benefit of having a consistent process that is applied across different practice change initiatives to help the team understand the steps:

“It [previous practice change initiative] was a good exercise to go through with the staff…when it came time to launch our next project, they’re understanding it’s the same—we’re going to follow the same model.” (Participant 12)

Another nine participants (41%) stated that their organization did not use a formal process or framework to guide the change process, with one participant stating they “fly by the seat of [their] pants” (Participant 2). The remaining participants (*n* = 4, 18%) stated their organizations do use a formal framework to guide practice changes, but they were not able to name it, as expressed by one director:

“I’m sure if you talked to somebody else, they could tell you what our actual change management tool is that we use. I just don’t have a good handle on that this morning, but it’s the basic principles that everyone else uses.” (Participant 4)

#### Engagement level

Five participants (23%) gave examples that indicated their level of engagement with point-of-care staff was meant to *inform* staff of the changes. This tended to be one-way communication from the working group to point-of-care staff about what decision was made and how it will be implemented. For instance, when asked if clinical staff offer their opinions on practice changes, one manager stated: “No, I don’t think so. We just made an executive decision around what we thought would help” (Participant 18).

Over three-quarters of participants (*n* = 17, 77%) provided examples of two-way exchanges with point-of-care staff during the implementation process. Participants most frequently provided examples of how they *consulted* with point-of-care staff (*n* = 12, 55%), for example, by asking for suggestions on what practice changes to prioritize or soliciting input on how the change process is working. These consultations occurred through formal ticket systems (i.e., staff submit ideas and suggestions), meetings and huddles, emails, and informal conversations.

Other participants (*n* = 6, 27%) described *involving* point-of-care staff (and in one case, patient advisors) in the change process through their involvement in unit committees and councils, as described by one participant:

“We have a patient and family-centered care steering committee, which is comprised of both hospital employees and patient advisors, and we solicit their input quite frequently with different actions that we need to take.” (Participant 13)

Two participants (9%) described *collaborating* with point-of-care staff by including them in the core implementation working group as equal partners in the process:

“They’re [clinical staff] part of the working group, so they participate in the change. They contribute in terms of the root cause identification, in terms of ideas, problem solving, what do they want to try, what do they think will work, what are the barriers. All of that, they’re involved in it.” (Participant 10)

## Discussion

In this study we aimed to explore how maternal-newborn hospital teams implement practice changes in their units. We learned about the usual implementation processes, focusing on what steps teams do and do not take. By comparing the described steps to an implementation framework, the Implementation Roadmap [36], we identified which steps were most frequently discussed (e.g., identifying a problem), which were less frequently discussed (e.g., selecting and adapting evidence, evaluation), and which were discussed frequently but not optimally (e.g., barriers assessment). We identified many strengths, including efforts to work through varied implementation steps, the depth of experiential knowledge, and efforts to engage point-of-care staff. By noting gaps in the implementation process, we identified potential areas where further capacity development and support may be needed.

Across the 22 sites, only one participant described all seven steps, with most describing four or less steps, potentially signaling that sites’ implementation processes are not comprehensive. Although we do not know what the sites’ implementation outcomes were (and therefore cannot make inferences about the effectiveness of the different approaches), our previous work identified that teams who were successful in their practice change initiatives were more likely to have used a formal framework (like the Implementation Roadmap) to guide their implementation process [39]. Furthermore, we identified variability across the sites regarding which implementation steps are taken. This is consistent with other studies that have reported differences in what, when, and how implementation steps are taken [40, 41]. There are several potential explanations for this variability. First, we expect that the nature of the change (e.g., size, complexity) may influence the number of steps that teams take, with smaller, simpler changes resulting in fewer steps taken. Second, the urgency of a change may prevent some steps from being taken (e.g., field-testing), such as when units are required to implement changes immediately due to organizational or provincial mandates (as we recently observed during the COVID-19 pandemic). Third, it is likely that the education and training experience of the implementation leader influences the process used. In our study, participants were predominantly nursing leaders who would have been trained in nursing clinical practice. Despite nurses frequently being tasked with improvement work, implementation practice and quality improvement are not typically included in nursing education programs and there are few opportunities for ongoing professional development in these areas [42, 43]. There is a need to better equip nurses with implementation science knowledge and skills to better position them to translate evidence-informed practices into care [44].

Some of the most frequently identified implementation steps were identifying a problem and best practice, assembling local evidence, and monitoring and evaluation. While it is promising to see so many sites engaging in these steps, it is important to consider how access to the BORN dashboard and registry may have contributed to these high numbers, and thus not necessarily be reflective of what is occurring in the broader maternal-newborn system outside of Ontario. The dashboard facilitates identification of a problem by alerting teams with a colored signal (red or yellow) when best practice is not being met; it assists with learning about current practice by allowing users to drill down into specific cases to explore factors that may be driving the observed rates; and it allows users to monitor changes over time by observing changes in their rates and colored signals [23, 45]. Other settings may not have access to a similar data system that has been designed to facilitate and improve care; rather, many teams rely on data systems designed for collecting data for clinical and administrative purposes, rather than monitoring and evaluation purposes [46, 47]. While our findings speak to the value of a dashboard for facilitating specific steps in the implementation process, our findings also highlight the need for teams to actively engage in implementation steps *beyond* using a dashboard. For instance, although many participants reported monitoring their dashboard (a largely passive activity), no participants described developing an evaluation plan beforehand and only one participant described undertaking a process and impact evaluation. More attention to active evaluation planning, consideration of broader outcomes (e.g., implementation outcomes, service outcomes, and client outcomes [48]), and resources to support evaluation are needed to better assess the effect of the practice change initiatives.

Although assessing barriers was one of the most frequently mentioned steps, study participants rarely described a comprehensive or theory-based approach, with some relying on experiential knowledge of barriers acquired through past projects. While the application of tacit knowledge is particularly useful in familiar situations [49], each practice change initiative is unique and relying on knowledge gained through past projects alone risks missing opportunities to learn about other relevant, current factors. In addition, few participants described selecting their implementation strategies based on the identified barriers or evidence. This challenge has been described elsewhere, with teams selecting strategies based on familiarity or the ISLAGIATT (“it seemed like a good idea at the time”) principle [50, 51]. While participants provided some examples of implementation strategies, it is important to note that this list is not exhaustive compared to the wide-ranging implementation strategies identified in the literature [52]. In our study, the most frequently identified implementation strategies were educational in nature, which aligns with previous literature [53, 54]. However, the barriers to practice change are often multi-factorial (e.g., at the individual, interpersonal, organizational, and system level), going beyond individual knowledge deficits. This requires implementation strategies that are tailored to the change being implemented, the identified multi-level barriers, and the implementation context [55]. Given there is evidence to suggest that tailoring implementation strategies to identified barriers can positively influence practice changes [56], there are opportunities to build further capacity in this area.

Less than half of participants named a process or framework that they use to guide the implementation process. Several study participants stated they used a framework or process but could not name it. Of those that did identify a process or framework, no one identified a comprehensive implementation framework (e.g., planned action framework [18]) that guided the full implementation process. Unsurprisingly, the most frequently identified processes and frameworks were grounded in quality improvement approaches (e.g., Lean, PDSA). Recently, there has been increased interest in the intersection between quality improvement and implementation science, with calls for the two complementary fields to better align [57, 58]. Adding implementation science to existing quality improvement approaches may have several benefits including an increased emphasis on using evidence-informed practices and a focus on applying theory-driven and systemic approaches to assessing determinants of practice and selecting implementation strategies [36]. We assert that implementation science can enhance (not replace) these existing quality improvement approaches and tools, providing a systematic and comprehensive approach for teams.

We identified examples of how teams engaged point-of-care staff to varying degrees in the implementation process, with most providing examples of two-way exchanges between the implementation working group and staff. Governance structures such as unit councils were identified as a means to facilitate this engagement. The COVID-19 pandemic may have resulted in changes to shared governance, with clinical priorities quickly shifting and “nonessential” activities such as council meetings sometimes suspended [59]. Because our data were collected pre-pandemic, it is unknown what shared governance structures remain in place and how this may have changed staff engagement. Our future work will explore the existing shared governance infrastructure and how it is used to facilitate engagement of point-of-care staff in the implementation process. While our study provided many examples of how point-of-care staff are engaged in practice changes, only one study participant described engaging patients or families and six described using patient education and engagement as an implementation strategy. Given the limited examples of engaging patients in the working group itself, there remain opportunities for earlier partnership with patients in the implementation process. Indeed, patients and caregivers can contribute meaningfully to the implementation process and can be a powerful motivator for changes [60].

### Limitations and strengths

We acknowledge this study has several limitations, many of which are inherent to conducting a secondary analysis. The interview questions were not designed to probe for the different Implementation Roadmap steps. The results need to be interpreted with caution; a participant not describing a step may in fact reflect a lack of precision in the interview questions, rather than an indication that the participant did not do it, or lacks the knowledge or skills to do it. Conversely, a participant stating they completed a step does not necessarily mean it was actually completed (or completed optimally). In addition, implementation science continues to grow yearly, and it is possible that at the time of the interviews, participants may not have had access to the same implementation language to articulate the Implementation Roadmap steps. However, implementation science has not been well translated to practice-based settings [9, 10, 43], and so this challenge would likely remain if the interviews were conducted today. To mitigate this challenge, we were liberal with our coding and coded according to participants’ descriptions, regardless of the specific terms used.

Next, our results may be influenced by social desirability bias, whereby participants shared information they perceived as socially acceptable, rather than sharing information that reflects their true reality [61]. For instance, some participants may have attempted to describe a more thorough implementation process than is actually used in practice. Brief or vague answers may be an indication of social desirability tendencies [61]; we were therefore attentive to this in our analysis, identifying where participants provided short answers without any elaboration on when or how the step is actually performed and highlighted this in our results (e.g., barriers assessment). However, social desirability was likely not an issue across all participants, as some did explicitly acknowledge their lack of awareness or completion of some steps.

Finally, it is important to acknowledge that at the time of analysis, the data were eight years old and these results may not reflect implementation practice in maternal-newborn hospitals today. To mitigate this limitation, each member of our research team was involved in interpreting the data, many of whom are clinicians embedded in maternal-newborn care. Based on our collective experience, and the knowledge that practice changes slowly [62], these findings would likely still ring true today. These results are being used to develop a survey to distribute to all Ontario maternal-newborn hospital units to learn about what Implementation Roadmap steps teams are currently taking, their confidence completing them, and their perception of their importance. The results we report here are informing the development of survey questions to probe identified gaps and to tailor the question wording to align with local language. The upcoming survey will complement this qualitative secondary analysis by providing updated data from a wider sample of hospitals, allowing us to better understand what gaps and needs remain.

Conducting a secondary analysis also offered several strengths. Given the current demands on our health system and its leaders, we may not have been able to enroll the same number of study participants in today’s conditions. Given the sufficient fit between the original dataset and our question, conducting a secondary analysis eliminated the participant burden that would have been required to collect new qualitative data from busy clinicians and administrators [24]. Another strength of our study was the application of a recent evidence-informed framework (Implementation Roadmap [36]) that synthesizes theoretical and experiential knowledge in implementation science and practice, allowing us to interpret the data in a new light and identify future areas for research and practical support. Finally, our study makes a unique contribution to the literature by describing and comparing the implementation approaches of many maternal-newborn teams. With data on 22 sites (about one-quarter of birthing hospitals in the province), our sample provides insight into the implementation processes of diverse teams, highlighting commonalities and differences. These insights serve as potential areas to focus future implementation capacity-building efforts in maternal-newborn health services.

## Conclusion

Overall, we observed variability in the reported implementation processes used by 22 maternal-newborn hospital units. While participants provided many examples of steps and activities they use to implement practice changes, we identified several areas where teams may need additional support. These results provide a foundation for future work to explore current implementation practice in maternal-newborn hospitals and will inform the development of tailored practice change resources, informed by implementation science, for maternal-newborn healthcare providers and leaders.

### Supplementary Information

**Additional file 1.** COREQ Reporting Checklist.

**Additional file 2.** Semi-structured interview guide.

## Data Availability

The dataset used in this secondary analysis (i.e., interview transcripts) are not publicly available due to them containing information that could compromise research participant privacy/consent, but they are available from the corresponding author on reasonable request.

## Abbreviations

BFI: Baby Friendly Initiative
BORN: Better Outcomes Registry & Network Ontario
MORE^OB^: Managing Obstetrical Risk Efficiently
PDCA: Plan-Do-Check-Act
PDSA: Plan-Do-Study-Act
QI: Quality improvement
RNAO: Registered Nurses’ Association of Ontario
SOGC: Society of Obstetricians and Gynecologists of Canada
SWOT: Strengths, Weaknesses, Opportunities, Threats
