# Supplementary material for: Planning and implementing practice changes in Ontario maternal-newborn hospital units: a secondary qualitative analysis
Source: BMC Pregnancy Childbirth. 2023 Oct 17;23:735. doi: 10.1186/s12884-023-06042-1 (PMC10583424; doi:10.1186/s12884-023-06042-1)
Supplement: Supplementary file 1 — Additional file 1. COREQ Reporting Checklist. [file 12884_2023_6042_MOESM1_ESM.docx]

**Additional File 1: Completed reporting checklist**

*This is a supplemental file to a full manuscript published in BMC Pregnancy and Childbirth. For full copyright and citation information see https://doi.org/10.1186/s12884-023-06042-1*

**Consolidated criteria for reporting qualitative research (COREQ) [25]**

|  | **Item**  **#** | **COREQ Item and Guide questions / description** | **Reported on page #** |
| --- | --- | --- | --- |
| **Domain 1: Research team and reflexivity** | | | |
| Personal characteristics | 1 | Interviewer/facilitator: Which author/s conducted the interview or focus group? | pp. 7-8 |
|  | 2 | Credentials: What were the researcher’s credentials? *e.g. PhD, MD* | p. 8 |
|  | 3 | Occupation: What was their occupation at the time of the study? | p. 8 |
|  | 4 | Gender: Was the researcher male or female? | p. 8 |
|  | 5 | Experience and training: What experience or training did the researcher have? | p. 8 |
| Relationship with participants | 6 | Relationship established: Was a relationship established prior to study commencement? | p. 8 |
|  | 7 | Participant knowledge of the interviewer: What did the participants know about the researcher? *e.g. personal goals, reasons for doing the research* | - |
|  | 8 | Interviewer characteristics: What characteristics were reported about the interviewer/facilitator? *e.g. Bias, assumptions, reasons and interests in the research topic* | pp. 7-8  p. 11 |
| **Domain 2: Study design** | | | |
| Theoretical framework | 9 | Methodological orientation and theory: What methodological orientation was stated to underpin the study? e.g. grounded theory,  discourse analysis, ethnography, phenomenology, content analysis | p. 7 |
| Participant selection | 10 | Sampling: How were participants selected? *e.g. purposive, convenience, consecutive, snowball* | p. 7 |
|  | 11 | Method of approach: How were participants approached? *e.g. face-to-face, telephone, mail, email* | p. 7 |
|  | 12 | Sample size: How many participants were in the study? | p. 7 |
|  | 13 | Non-participation: How many people refused to participate or dropped out? Reasons? | p. 7 |
| Setting | 14 | Setting of data collection: Where was the data collected? *e.g. home, clinic, workplace* | p. 8 |
|  | 15 | Presence of non-participants: Was anyone else present besides the participants and researchers? | p. 8 |
|  | 16 | Description of sample: What are the important characteristics of the sample? *e.g. demographic data, date* | p. 7  p. 12 (Table 1) |
| Data collection | 17 | Interview guide: Were questions, prompts, guides provided by the authors? Was it pilot tested? | Additional file 2 |
|  | 18 | Repeat interviews: Were repeat interviews carried out? If yes, how many? | - |
|  | 19 | Audio/visual recording: Did the research use audio or visual recording to collect the data? | p. 8 |
|  | 20 | Field notes: Were field notes made during and/or after the interview or focus group? | p. 9 |
|  | 21 | Duration: What was the duration of the interviews or focus group? | P. 8 |
|  | 22 | Data saturation: Was data saturation discussed? | p. 7 |
|  | 23 | Transcripts returned: Were transcripts returned to participants for comment and/or correction? |  |
| **Domain 3: Analysis and findings** | | | |
| Data analysis | 24 | Number of data coders: How many data coders coded the data? | pp. 10-11 |
|  | 25 | Description of the coding tree: Did authors provide a description of the coding tree? | p. 13 (Figure 2) |
|  | 26 | Derivation of themes: Were themes identified in advance or derived from the data? | p. 9  p. 11 |
|  | 27 | Software: What software, if applicable, was used to manage the data? | p. 9 |
|  | 28 | Participant checking: Did participants provide feedback on the findings? | p. 11 |
| Reporting | 29 | Quotations presented: Were participant quotations presented to illustrate the themes / findings? Was each quotation identified? *e.g. participant number* | pp. 14-23 |
|  | 30 | Data and findings consistent: Was there consistency between the data presented and the findings? | pp. 13-23 |
|  | 31 | Clarity of major themes: Were major themes clearly presented in the findings? | pp. 13-23 |
|  | 32 | Clarity of minor themes: Is there a description of diverse cases or discussion of minor themes? | pp. 13-23 |

Tong A, Sainsbury P, Craig J. Consolidated criteria for reporting qualitative research (COREQ): A 32-item checklist for interviews and focus groups. Int J Qual Heal Care. 2007;19:349–57. doi: 10.1093/intqhc/mzm042
