# Supplementary material for: Planning and implementing practice changes in Ontario maternal-newborn hospital units: a secondary qualitative analysis
Source: BMC Pregnancy Childbirth. 2023 Oct 17;23:735. doi: 10.1186/s12884-023-06042-1 (PMC10583424; doi:10.1186/s12884-023-06042-1)
Supplement: Supplementary file 2 — Additional file 2. Semi-structured interview guide. [file 12884_2023_6042_MOESM2_ESM.docx]

**Additional File 2: Semi-Structured Interview Guide**

*This is a supplemental file to a full manuscript published in BMC Pregnancy and Childbirth. For full copyright and citation information see https://doi.org/10.1186/s12884-023-06042-1*

1. Do you access the dashboard yourself? If yes, what features do you find the most useful/least useful?

*[If no, proceed to Question 3]*

**Probes:**

- 1. Dashboard visual data displays such as the dashboard homepage (stoplight signals)?
  2. Dashboard reports and sub-reports (data tables with numbers, rates confidence intervals, comparator data)?
  3. Dashboard audit features and functionality (sub-report that links with the record level data entered into the BORN Information System at your site; missing data reports)?
  4. Key performance indicator definitions (details provided below each of the data tables to clarify inclusion/exclusion criteria for each key performance indicator)?
  5. Knowledge-to-Action Evidence Summaries (evidence to support each key performance indicator available on the BORN website)?

1. Who else accesses the dashboard? Do you think they share the same sentiment in terms of what they find most and least useful about the dashboard?
2. If someone else in your organization accesses the dashboard for you, can you comment on the features they find most or least useful?
3. How is data entered into the BORN Information System at your site (e.g. manual versus electronic medical record, clerk versus clinicians, retrospective versus real-time)?
4. What problems or barriers have you (or your delegate) encountered using the dashboard?

**Probes:**

- 1. Computer issues with network access to the BORN Information System (e.g. slow system)?
  2. Lack of resources (e.g. computer stations) and time to enter the data?
  3. Availability of appropriate staff to enter data and run the reports?
  4. Staff attitudes, resistance, perceptions about the credibility of the data?
  5. Other priorities?

Thinking of a recent project (if there’s one using the dashboard key performance indicators, please use that one), can you tell me a bit about how you plan for this kind of practice change in your hospital? Are there specific structures or processes in place to support change?

**Probes:**

- 1. How did you know there was a practice issue that needed to be addressed? If the project relates to the dashboard, can you tell me how the key performance indicator was selected?
     - Organizational or provincial priorities?
     - Directives from the Chief of Obstetrics or other administrator?
     - Degree of difficulty of the practice change issue?
  2. How did you know there was a practice issue that needed to be addressed?
  3. Did you use a framework to guide the change process (e.g. Plan-Do-Study-Act, Knowledge-to-Action cycle, etc.)?
  4. Were barriers to change assessed?
  5. How were the interventions /strategies to solve the evidence-practice gap selected?
  6. Was the change monitored?
  7. What is the current status of this process change?
  8. Were there specific interventions used to ensure sustainability?
  9. What was the outcome of that project?

**Thinking of the project you just described to me…**

1. Who was involved in the change process?

**Probes:**

- 1. Who was the designated leader responsible for the change?
  2. How did the leader(s) facilitate change?
- Sent memos, scheduled workshops, gathered the team to discuss the issue
- Do they normally take a proactive or reactive approach to change?
  1. Champions with responsibility to facilitate change?
  2. Quality or departmental committees that met regularly to discuss practice change issues?
  3. Someone responsible for the different components of the change process (e.g. evidence-practice gap identification, barriers assessment, implementation, evaluation etc.)
  4. Who normally sees the dashboard data? Are your clinicians informed about the dashboard key performance indicators?

1. What about *team involvement* in the change process? How did your clinical staff contribute to the change process?

**Probes:**

1. Managers solicited opinions of clinical staff regarding practice change issues?
2. Discussed practice change issues at team meetings?
3. The team routinely works together to formulate action plans and monitor performance?
4. Were there different *inter-professional* *perspectives* evident for this practice issue? If yes, was there a formal process used to work through the inter-professional differences? Please describe the process.

**Probes:**

1. If there was a practice issue that touched both physician and midwifery practice, for example, how were decisions made about priorities or strategies for change?
2. Would you say decisions were driven by power, structures, or personalities around the table or was there a specific process used to resolve differences?
3. What *organizational supports* are available or have you used to help team members address issues related to practice change? [e.g. dashboard key performance indicator or other practice issues]

**Probes:**

1. Internal resources/supports within your organization
   - Champions or other resources/supports you have control over?
   - Formal mechanism to obtain feedback related to proposed change?
   - What resources or supports would be helpful to assist you with practice change?
2. External resources/supports from other organizations
   - Access to BORN Coordinators?
   - BORN webinars and training?
   - Resources produced by other organizations (e.g., Provincial Council for Maternal and Child Health (PCMCH) webinars)?
3. [**Only for those with a dashboard example**] Is this change process the same for other projects in your hospital or is there something special about the dashboard?
   1. What about for other key performance indicators?
4. Though we have attempted to be thorough in our questions, is there anything that perhaps we didn’t cover that you think would be important to note in terms of use of the dashboard or the key performance indicator data?
